# Patient-Specific MRI-Based Right Ventricle Models Using Different Zero-Load Diastole and Systole Geometries for Better Cardiac Stress and Strain Calculations and Pulmonary Valve Replacement Surgical Outcome Predictions

**DOI:** 10.1371/journal.pone.0162986

**Published:** 2016-09-14

**Authors:** Dalin Tang, Pedro J. del Nido, Chun Yang, Heng Zuo, Xueying Huang, Rahul H. Rathod, Vasu Gooty, Alexander Tang, Zheyang Wu, Kristen L. Billiar, Tal Geva

**Affiliations:** 1 School of Biological Science & Medical Engineering, Southeast University, Nanjing, China; 2 Mathematical Sciences Department, Worcester Polytechnic Institute, Worcester, MA, United States of America; 3 Department of Cardiac Surgery, Boston Children’s Hospital, Department of Surgery, Harvard Medical School, Boston, MA, United States of America; 4 China Information Tech. Designing & Consulting Institute Co., Ltd., Beijing, China; 5 School of Mathematical Sciences, Xiamen University, Xiamen, Fujian, China; 6 Department of Cardiology, Boston Children's Hospital, Department of Pediatrics, Harvard Medical School, Boston, MA, United States of America; 7 Department of Biomedical Engineering, Worcester Polytechnic Institute, Worcester, MA, United States of America; 8 Department of Surgery, University of Massachusetts Medical School, Worcester, MA, United States of America; Semmelweis Egyetem, HUNGARY

## Abstract

**Background:**

Accurate calculation of ventricular stress and strain is critical for cardiovascular investigations. Sarcomere shortening in active contraction leads to change of ventricular zero-stress configurations during the cardiac cycle. A new model using different zero-load diastole and systole geometries was introduced to provide more accurate cardiac stress/strain calculations with potential to predict post pulmonary valve replacement (PVR) surgical outcome.

**Methods:**

Cardiac magnetic resonance (CMR) data were obtained from 16 patients with repaired tetralogy of Fallot prior to and 6 months after pulmonary valve replacement (8 male, 8 female, mean age 34.5 years). Patients were divided into Group 1 (n = 8) with better post PVR outcome and Group 2 (n = 8) with worse post PVR outcome based on their change in RV ejection fraction (EF). CMR-based patient-specific computational RV/LV models using one zero-load geometry (1G model) and two zero-load geometries (diastole and systole, 2G model) were constructed and RV wall thickness, volume, circumferential and longitudinal curvatures, mechanical stress and strain were obtained for analysis. Pairwise T-test and Linear Mixed Effect (LME) model were used to determine if the differences from the 1G and 2G models were statistically significant, with the dependence of the pair-wise observations and the patient-slice clustering effects being taken into consideration. For group comparisons, continuous variables (RV volumes, WT, C- and L- curvatures, and stress and strain values) were summarized as mean ± SD and compared between the outcome groups by using an unpaired Student t-test. Logistic regression analysis was used to identify potential morphological and mechanical predictors for post PVR surgical outcome.

**Results:**

Based on results from the 16 patients, mean begin-ejection stress and strain from the 2G model were 28% and 40% higher than that from the 1G model, respectively. Using the 2G model results, RV EF changes correlated negatively with stress (r = -0.609, P = 0.012) and with pre-PVR RV end-diastole volume (r = -0.60, P = 0.015), but did not correlate with WT, C-curvature, L-curvature, or strain. At begin-ejection, mean RV stress of Group 2 was 57.4% higher than that of Group 1 (130.1±60.7 vs. 82.7±38.8 kPa, P = 0.0042). Stress was the only parameter that showed significant differences between the two groups. The combination of circumferential curvature, RV volume and the difference between begin-ejection stress and end-ejection stress was the best predictor for post PVR outcome with an area under the ROC curve of 0.855. The begin-ejection stress was the best single predictor among the 8 individual parameters with an area under the ROC curve of 0.782.

**Conclusion:**

The new 2G model may be able to provide more accurate ventricular stress and strain calculations for potential clinical applications. Combining morphological and mechanical parameters may provide better predictions for post PVR outcome.

## 1. Introduction

Accurate ventricle stress and strain calculations are of fundamental importance for cardiovascular research and investigations. From mechanical point of view, zero-stress ventricle geometry information is required for its stress/strain calculations. Ventricle modeling, especially ventricle active contraction modeling based on in vivo data, is extremely challenging because of complex ventricle geometry, dynamic heart motion and active contraction and relaxation where the reference geometry (zero-stress geometry) changes constantly in a cardiac cycle. As a first-order approximation, an approach using two zero-load geometries (2G) is proposed to model ventricle cardiac motion: one zero-load ventricle geometry is used to model the diastole phase where sarcomere has its relaxed zero-stress length, another zero-load ventricle geometry is used to model the systole phase where sarcomere has its contracted zero-stress length (therefore the zero-load systole geometry is smaller than the zero-load diastole geometry). Essentially, we are using two models to model the cardiac cycle to handle the active contraction and relaxation which are caused by zero-stress sarcomere length changes. It should be noted that zero-stress and zero-load are two different concepts. Zero-load geometries are used as an approximation since zero-stress state is really hard to get, and zero-load geometries are what we need for model construction purposes. More details are given later.

A brief review of ventricle modeling is given in the Discussion section. Active contraction is caused by sarcomere shortening which leads to increased strain and stress. A brief description is given with the use of some related terminologies: a) at the beginning of active contraction, the zero-stress sarcomere length is shortened in a very short time duration while ventricle volume has no change (isovolumic); b) since the ventricle volume does not change, while there are local SL variations and ventricle shape changes [1], the average in vivo sarcomere length under pressure (referred to as in vivo SL) may not change much when the zero-stress sarcomere length shortens (not visible in vivo) which leads to ventricle strain increase; c) the increased strain leads to the “added” stress, equivalent to the active tension in models in [2,3]. The same could be said about “active relaxation” which happens at the end of systole when the zero-stress systolic sarcomere length changes back to its zero-stress diastolic length.

The active-tension models in [2,3] is well-accepted which adds an active stress term to ventricle total stress. Since it does not change its reference geometry, strain calculation was still based on one geometry and does not reflect the effect caused by zero-stress SL changes. A new modeling approach using two different zero-load geometries (diastole and systole) was introduced in this paper to properly model active contraction and relaxation and provide ventricle diastole and systole stress and strain calculations based on their respective zero-load geometries. 2G models were constructed for 16 patients with repaired Tetralogy of Fallot (TOF) (data from a clinical trial and available for our research) and results were compared with our previously published one-geometry (1G) models [4–7]. The new morphological and stress/strain results from the new 2G models were used to identify potential predictors for post pulmonary valve replacement (PVR) outcome for ToF patients.

## 2. Methods

### 2.1. Modeling active contraction and expansion by using different zero-load diastole and systole geometries

As a first order approximation, the right ventricle cardiac cycle can be divided into 4 phases involving two different RV zero-stress geometries (diastole and systole). A short description of the 4 phases is given below since this is the base for our 2-geometry models:

#### Phase 1

Filling (diastole phase). The right ventricle starts with its minimum volume under minimum pressure with minimum stress and strain. One zero-load geometry (diastole geometry) is used for this phase, corresponding to diastole zero-stress sarcomere length (SL). It should be noted that zero-stress status is a concept for stress/strain calculations. It is not observable in a living heart under in vivo conditions. At beginning-of-filling, tricuspid valve opens; RV volume increases, pressure increases, in vivo SL expands; strain and stress increases. Phase 1 ends when RV reaches its maximum volume under end-diastole pressure (denoted by P-dia) which is lower than the maximum pressure condition.

#### Phase 2

Isovolumic contraction: Both tricuspid and pulmonary valves are closed; RV volume has no change; zero-stress SL shortens (changing from diastole zero-stress length to systole zero-stress length); however, this sarcomere shortening is not physically observable. Roughly, average in vivo SL does not change much (small local SL changes are possible) since RV volume does not change. So zero-stress SL shortening leads strain and stress increase (This is similar to the active tension in other models, but our model have both strain and stress increase); increased stress pushes pressure to maximum. This phase is short. This phase involves dynamic change of zero-stress sarcomere length which is very difficult to implement. It was skipped in our model.

#### Phase 3

Ejection (systole phase): This phase starts from max volume, pressure, stress and strain. One zero-load geometry (for systole phase) is used for this phase, corresponding to systole zero-stress SL. At begin-systole (referred to as begin-ejection as well), pulmonary valve opens up and ejection starts; RV volume drops; in vivo SL shortens and strain decreases; pressure drops; stress drops. At end-systole (end-ejection), RV volume reaches its minimum, pressure drops to the end-systole pressure denoted as P-sys, which is greater than minimum pressure. Pressure will continue to drop in Phase 4 when systole zero-stress SL changes to diastole zero-stress SL.

#### Phase 4

Isovolumic relaxation: Pulmonary valve closes (both valves closed); zero-stress SL relaxes from systole zero-stress length to diastole zero-stress length (non-contracted length); similar to the comments made in Phase 2, roughly, average in vivo SL does not change much since volume does not change; zero-stress SL relaxation leads to strain and stress decreases; pressure drops to minimum. This phase is short. It was also skipped in our model.

Our 2G model includes the diastole and systole phases described above with their respective zero-load ventricle geometries reconstructed from patient-specific MRI data. In fact, the 2G model includes two sub-models, systole and diastole models, each with its own zero-load geometry and pressure conditions. They are based on the same CMR data with continuous volume variation in a cardiac cycle. The two isovolumic phases were omitted from our model. So stress and strain values have discontinuities going between the two phases. RV volume, pressure, stress and strain achieve their minima and maxima at begin-diastole (BD) and begin-systole (BS), respectively. End-diastole and end-systole pressure, stress and strain are also available from our 2G model which were not available from our 1G models [4–7] since end-diastole and begin-systole were made identical in 1G models, as well as end-systole and begin-diastole, respectively. Therefore, 2G model represents an improvement over 1G model also in that regard.

### 2.2. Data acquisition and 3D geometry reconstruction

**Fig 1:**
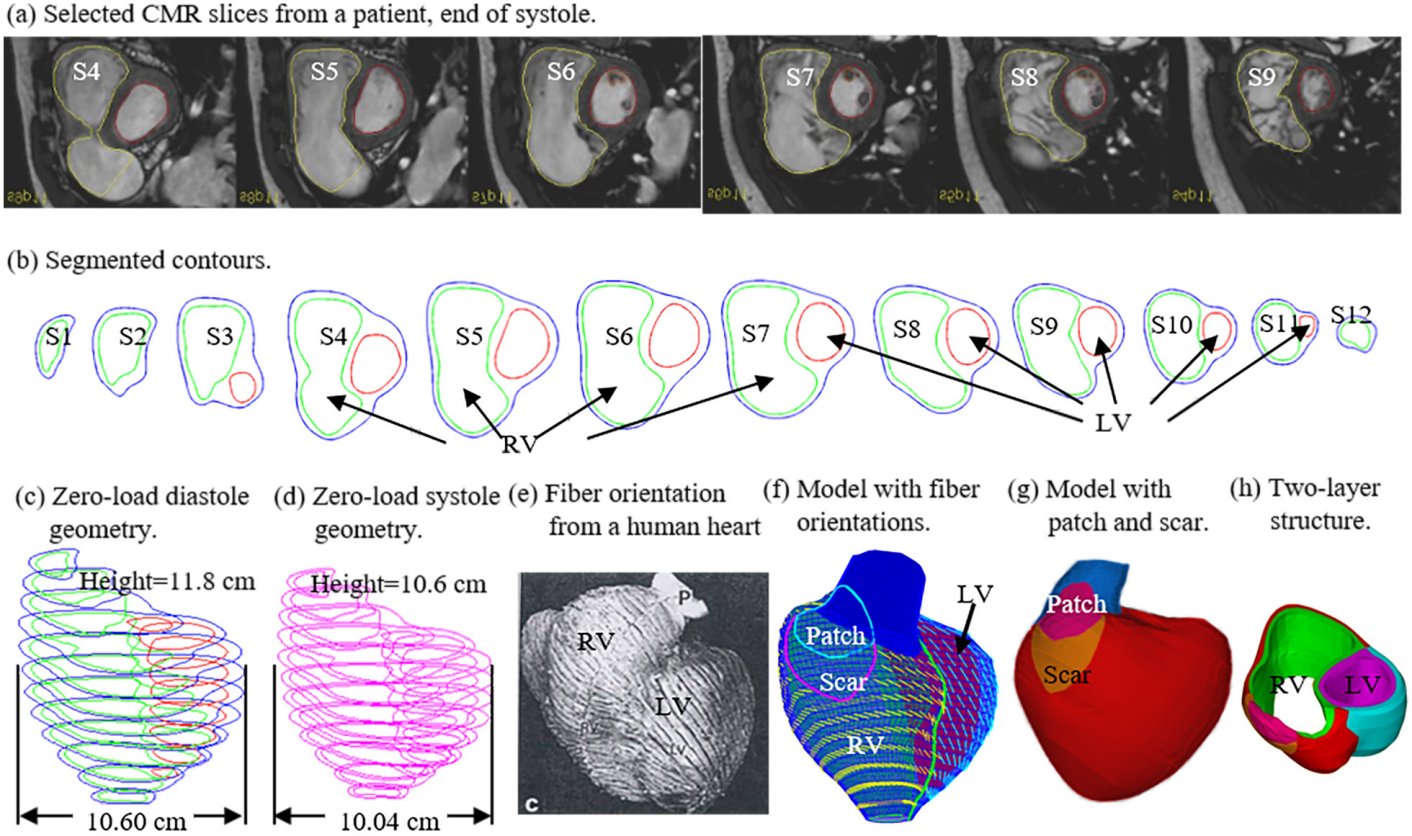
CMR-based model construction process and zero-load diastole and systole geometries. (a) Selected CMR slices from a patient, end of systole; (b) segmented contours; (c) zero-load diastole geometry; (d) zero-load systole geometry; (e) fiber orientation from a human heart; (f) model with marked fiber orientations; (g) model with patch and scar; (h) two-layer structure.

The Boston Children’s Hospital Committee on Clinical Investigation approved the study. The Boston Children’s Hospital IRB approval number is: IRB-CRM09-04-0237. Written informed consent was obtained from participants. Cardiac magnetic resonance (CMR) data before and 6 months after PVR surgery were obtained from 16 patients (8 male, 8 female, mean age 34.5) who were previously enrolled in the RV surgical remodeling trial [8]. For this analysis, we selected the 8 best (Group 1) and 8 worst (group 2) responders based on their change in RV EF from pre- to post-PVR. RV EF was chosen due to its strong association with adverse clinical outcomes in patients with repaired TOF. Table 1 describes the basic patient data and RVEF from study cohort. Briefly, ventricular assessment was performed by an electrocardiographically-gated, breath-held steady state free precession cine CMR in ventricular short-axis planes (12–14 equidistant slices covering the atrioventricular junction through the apex). The pressure data were obtained from pre-PVR cardiac catheterization procedures. The use of CMR in evaluating RV function and size in clinical practice has been well established [9–10]. The resolution of the CMR technique used in our patients was 1.6 × 1.6 × 6–8 mm reconstructed to 1.0 × 1.0 × 6–8 mm (field of view 260 × 260 mm^2^; matrix 160 × 160 reconstructed to 256 × 256; 30 reconstructed frames per cardiac cycle). The valve and patch positions were determined with cine MR imaging, flow data, and delayed enhancement CMR to delineate location and extent of scar/patch, and were further verified by the surgeon (PJdN, 30 years of experience) who performed PVR for those patients. Ventricular volumes and function were measured by manual tracing of endocardial and epicardial borders on each short-axis steady-state free precession cine slices throughout the entire cardiac cycle. Analyses were performed using commercially available software (QMass, Medis Medical Imaging Systems, Leiden, the Netherlands). Simpson’s method was applied to calculate end-diastolic volume (EDV), end-systolic volume (ESV), EF, stroke volume, ventricular mass, and mass-to-volume ratio. Three-dimensional RV/LV geometry and computational meshes were constructed as previously described [4–7]. Fig 1 shows sample pre-operative CMR images from a TOF patient with segmented contour plots, reconstructed 3D zero-load diastole and systole geometries (explained in Section 2.3), a front view showing the patch, scar, and the RVOT. Fiber orientation and the two-layer model construction were also shown (Fig 1d–1g) [3,11]. Since patient-specific fiber orientation data were not available, data from the current literature was used in our models.

**Table 1:** Demographic and CMR data before and after PVR.

			Pre-PVR			Post-PVR			
Pt.	Sex	Age (y)	RV EDV (cm^3^)	RV ESV (cm^3^)	RV EF (%)	RV EDV (cm^3^)	RV ESV (cm^3^)	RV EF (%)	ΔEF (%)
P1	M	22.5	406.9	254.5	37.5	188.3	115.0	38.9	1.4
P2	F	38.5	328.8	196.0	40.4	168.3	106.0	37.0	-3.4
P3	M	47.7	408.8	254.8	37.7	327.2	212.3	35.1	-2.6
P4	M	50.0	364.6	239.5	34.3	220.0	150.9	31.4	-2.9
P5	F	42.0	323.3	177.8	45.0	222.2	113.3	49.0	4.0
P6	F	14.3	204.0	104.3	48.8	136.5	62.3	54.4	5.6
P7	F	15.3	193.7	105.1	45.7	177.9	84.8	52.3	6.6
P8	M	17.0	188.3	108.3	42.5	135.5	75.2	44.5	2.0
P9	F	56.9	385.1	184.6	52.1	216.4	142.6	34.1	-18.0
P10	M	11.6	204.2	121.3	40.6	156.3	106.0	32.2	-8.4
P11	M	43.5	665.1	464.0	30.2	391.0	332.4	15.0	-15.2
P12	M	54.1	334.8	170.8	49.0	306.2	177.6	41.0	-7.0
P13	F	49.5	277.2	151.3	45.4	244.8	145.9	40.4	-5.0
P14	M	17.8	365.0	178.0	51.2	292.1	170.2	41.7	-9.5
P15	F	44.6	299.0	186.0	37.8	218.7	163.0	25.5	-12.3
P16	F	45.3	571.1	371.3	35.0	398.7	312.6	21.6	-13.4
Mean ± SD		40.4 ±16.6	387.7 ±154.8	228.4 ±121.3	42.7 ±8.0	278.0 ±85.8	193.8 ±82.6	31.4 ±9.9	-11.1 ±4.4

Abbreviations: F, Female; M, male; EDV, end-diastolic volume; ESV, end-systolic volume; EF, ejection fraction.

### 2.3. A pre-shrink process to obtain no-load diastole and systole geometries

Under the *in vivo* condition, the ventricles were pressurized and the zero-load ventricular geometries were not known. In our model construction process, an iterative pre-shrink process was applied to the *in vivo* minimum volume ventricular geometry to obtain the two zero-load geometries so that when in vivo pressure was applied, the ventricle would regain its *in vivo* geometry. Shrinking is achieved by shrinking each slice (short-axis direction) by a shrinking rate and by reducing the slice distances (long-axis direction). However, if the slice was shrunk uniformly, the ventricle wall volume (the muscle) would become smaller, which should not happen. So the inner contour (inner wall of the ventricle) was shrunk more, the outer contour (ventricle outer wall) was shrunk a little less (rate was determined by volume conservation). To get the zero-load diastole geometry, we started with a 2% shrinkage, construct the model, and apply the minimum pressure to see if the pressurized RV volume matches the CMR data. If not, we adjust the shrinkage, re-made the model, pressurize it and check again. The process is repeated until RV volume matches CMR volume with error < 0.5%. For the zero-load systole geometry, assuming a 10–15% sarcomere shortening, we started with a 15% shrinkage. The same process was repeated until the pressurized RV volume under end-systole pressure (higher than the minimum pressure) matched the CMR volume data. LV geometries were handled in similar way, with proper shrinkages determined corresponding to LV pressure conditions.

### 2.4. Direct biaxial testing of human myocardium tissue material properties

Tissue mechanical properties are essential to computational ventricle models. However, human heart tissue material properties are not readily available from the literature. Based on the methods of Sacks and Choung [12–13] for canine hearts and informed by our previous biaxial testing [14] and the methods of Humphrey et al. and Novak et al. [15–16]. We generated the first complete multiaxial mechanical data set for ventricular tissues using a cadaveric human heart sample [7]. A detailed description of the custom planar biaxial testing device and method has been previously described [12,14]. It should be noted that the direct measurement of biaxial material data was used to verify that the modified Mooney-Rivlin model is able to fit the recorded stress-strain data. The parameter values in the Mooney-Rivlin model were adjusted for each patient to match CMR-measured volume data.

### 2.5. The anisotropic material models for RV tissues, fiber orientation and two-layer model construction

The governing equations for all material models were:

$$\rho v_{i,tt}=\sigma_{ij,j},\;i,j=1,2,3;sum\;over\;j,$$ (1)

$$\varepsilon_{ij}=(v_{i,j}+v_{j,i}+v_{\alpha ,i}v_{\alpha ,j})/2,\;i,j,\alpha =1,2,3,$$ (2)

$$p{|}_{RV}=p_{RV}\left(t\right),\;p{|}_{LV}=p_{LV}\left(t\right),$$ (3)

where **σ** is the stress tensor, **ε** is the strain tensor, **v** is displacement, and ρ is material density. The normal stress was assumed to be zero on the outer RV/LV surface and equal to the pressure conditions imposed on the inner RV/LV surfaces. Structure-only RV/LV models were used to optimize model computing time. These models provided RV volume, ejection fractions, and RV stress/strain values for analysis.

The RV and LV materials were assumed to be hyperelastic, anisotropic, nearly-incompressible and homogeneous. The patch and scar materials were assumed to be hyperelastic, isotropic, nearly-incompressible and homogeneous. The nonlinear Mooney-Rivlin model was used to describe the nonlinear anisotropic and isotropic material properties. The strain energy function for the isotropic modified Mooney-Rivlin model is given by Tang et al. [4–7]:

$$W=c_{1}\left(I_{1}-3\right)+c_{2}\left(I_{2}-3\right)+D_{1}\left[\text{exp}\left(D_{2}\left(I_{1}-3\right)-1\right)\right],$$ (4)

where I_1_ and I_2_ are the first and second strain invariants given by,

$$I_{1}=\sum C_{ii},\;I_{2}=\frac{1}{2}\left[I_{1}^{2}-C_{ij}C_{ij}\right],$$ (5)

**Fig 2:**
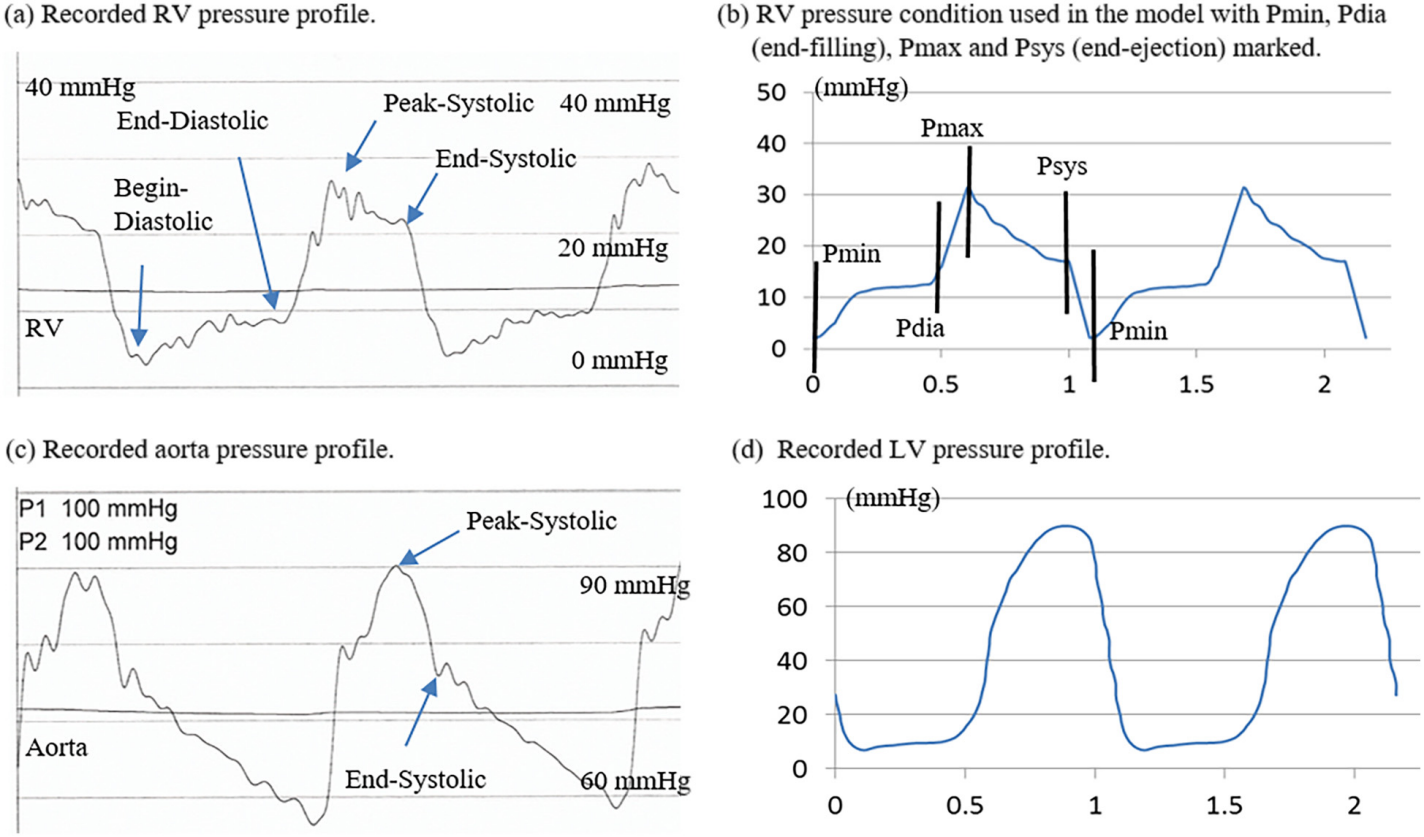
Recorded patient-specific pressure profiles and pressure conditions imposed on computational models. (a) Recorded RV pressure profile; (b) RV pressure condition used in the model with Pmin (begin-filling), Pdia (end-filling), Pmax, (begin-ejection) and Psys (end-ejection) marked; (c) recorded aorta pressure profile; (d) recorded LV pressure profile.

**Fig 3:**
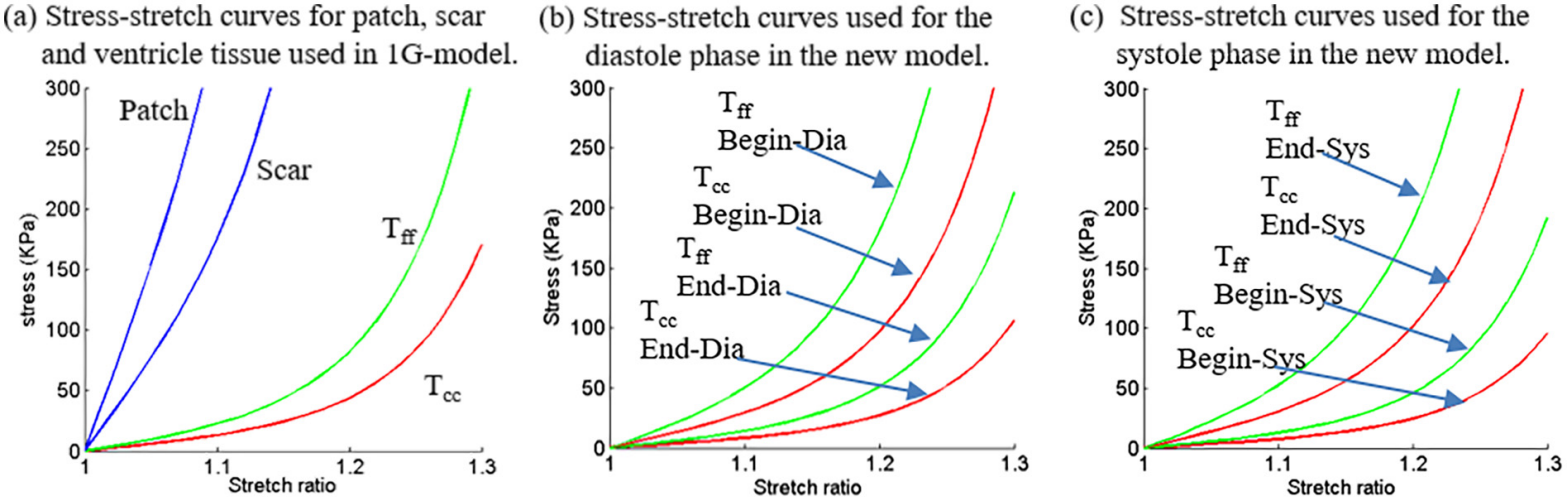
Material Stress-Stretch curves used for the new 2G and old 1G models. (a) Stress-stretch curves for patch, scar and ventricle tissue used in 1G model; (b) stress-stretch curves used for the diastole phase in the 2G model; (c) stress-stretch curves used for the systole phase in the 2G model. T_ff_: stress in fiber direction; T_cc_: stress in cross-fiber direction.

**Fig 4:**
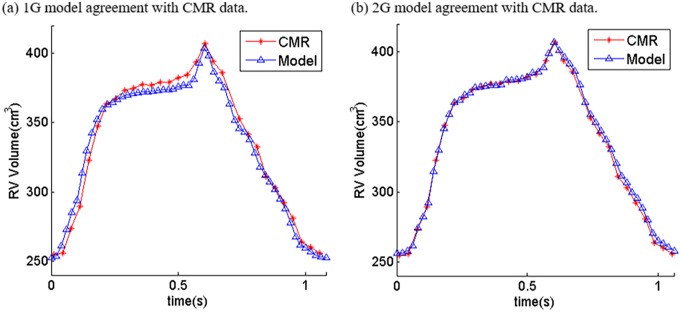
Computational volume results from 1G and 2G models. (a) 1G model agreement with CMR data; (b) 2G model agreement with CMR data.

C = [C_ij_] = X^T^X is the right Cauchy-Green deformation tensor, X = [X_ij_] = [∂x_i_/∂a_j_], (x_i_) is the current position, (a_i_) is the original position, c_i_ and D_i_ are material parameters chosen to match experimental or patient-specific CMR measurements. The strain energy function for the anisotropic modified Mooney-Rivlin model was obtained by adding an additional anisotropic term in Eq 4 (Tang et al. [5–7]):

$$W=c_{1}\left(I_{1}-3\right)+c_{2}\left(I_{2}-3\right)+D_{1}\left[\text{exp}\left(D_{2}\left(I_{1}-3\right)-1\right)\right]+\frac{K_{1}}{2K_{2}}\text{exp}\left[K_{2}{\left(I_{4}-1\right)}^{2}-1\right],$$ (6)

where *I*_4_ = C_ij_ (**n**_f_)_i_ (**n**_f_)_j_, C_ij_ is the Cauchy-Green deformation tensor, **n**_f_ is the fiber direction, K_1_ and K_2_ are material constants. With parameters properly chosen, it was shown that stress-strain curves derived from Eq 6 agreed very well with the stress-strain curves from the anisotropic (transversely isotropic) strain-energy function with respect to the local fiber direction given in McCulloch et al. [2]:

$$W=\frac{C}{2}\left(e^{Q}-1\right),$$ (7)

$$Q=b_{1}E_{ff}^{2}+b_{2}\left(E_{cc}^{2}+E_{rr}^{2}+E_{cr}^{2}+E_{rc}^{2}\right)+b_{3}\left(E_{fc}^{2}+E_{cf}^{2}+E_{fr}^{2}+E_{rf}^{2}\right),$$ (8)

where *E*_*ff*_ is fiber strain, *E*_*cc*_ is cross-fiber in-plane strain, *E*_*rr*_ is radial strain, and *E*_*cr*_, *E*_*fr*_ *and E*_*fc*_ are the shear components in their respective coordinate planes, *C*, *b*_*1*_, *b*_*2*_, and *b*_*3*_ are parameters to be chosen to fit experimental data. Even though two different zero-load geometries were used for diastole and systole phases, parameter values in 6–8 still needed to be adjusted to fit CMR-measured RV volume data. It should be noted that Eqs 7 and 8 were used because it is desirable to use local coordinate system to identify material parameters which are independent of fiber directions. Stress-stretch curves from our old one-geometry model and the new two geometry model for the patient given in Fig 1 are given in Fig 2. Imposed RV pressure conditions are given by Fig 3. Fig 4 shows good agreement between computational and CMR-measured volume data (error < 2%).

### 2.6. Fiber orientation

As patient-specific fiber orientation data was not available, we chose to construct a 2-layer RV/LV model and set fiber orientation angles using fiber angles given in Hunter et al. (see Fig 1) [3]. Fig 1 shows fiber orientations from a human heart and how the 2-layer RV/LV model was constructed (Fig 1(h)) [4–7].

### 2.7. Mesh generation and geometry-fitting technique for patient-specific CMR-based models

Because ventricles have complex irregular geometries with patch and scar tissue components, a geometry-fitting mesh generation technique was developed to generate mesh for our models. Fig 1(h) illustrates RV/LV geometry between 2 slices. Each slice was first divided into geometry-fitting areas called “surfaces”. The neighboring slices were stacked to form volumes. Using this technique, the 3D RV/LV domain was divided into multiple “subvolumes” to curve-fit the irregular ventricle geometry with patch as an inclusion. 3D surfaces, volumes and computational mesh were made manually, slice by slice. Mesh analysis was performed by decreasing mesh size by 10% (in each dimension) until solution differences in stress/strain predictions were less than 2%. The mesh was then chosen for our simulations.

### 2.8. Solution methods and simulation procedures

The unsteady periodic RV/LV computational models were constructed for the 16 patients and the models were solved by ADINA (ADINA R&D, Watertown, MA, USA) using unstructured finite elements and the Newton-Raphson iteration method. Simulations were carried out for 3 periods until the solutions became period and stress/strain distributions from the 3^rd^ period were extracted for analysis. Because stress and strain are tensors, for simplicity, maximum principal stress (Stress-P_1_) and strain (Strain-P_1_) were used as the representatives and referred to as stress and strain in this paper.

### 2.9. Data collection for statistical analysis

For each CMR data set, we divided each slice into 4 quarters, each with equal inner wall circumferential length. Ventricular wall thickness (WT), circumferential curvature (C-cur), longitudinal curvature (L-cur), and stress/strain were calculated at all nodal points (100 points/slice). The “slice” values of those parameters were obtained by taking averages of those quantities over the 100 points for each slice and saved for analysis. We checked the data for the proper assumptions of statistical methods. In particular, for statistical studies using the 16 patients (sample size n = 16), the assumption of normality was checked and satisfied. Pairwise T-test and Linear Mixed Effect (LME) model were used to determine if the differences from the new and old models were statistically significant, with the dependence of the pair-wise observations and the patient-slice clustering effects being taken into consideration [17]. For group comparisons, continuous variables (RV volumes, WT, C- and L- curvatures, and stress and strain values) were summarized as mean ± SD and compared between the outcome groups by using an unpaired Student t-test. Associations between pre-PVR RV parameters and the outcome (change in RV EF) were explored using Pearson correlation analysis. At the patient level (assuming independence), logistic regression analysis was used to identify pre-PVR parameters that best predicted the primary outcome—RV EF response to PVR. Prediction performance was evaluated based on 20 repeats of 2-fold cross-validation in order to stabilize the accuracy measurements for all combinations of these parameters [7,18]. Repeated cross-validation is a standard technique to reduce errors and improve prediction stability, especially when sample size is relatively small [19,20]. For each predictor (it can be a combination of the risk factors) and for each test run, the data set was divided randomly into two parts, one part (model fitting data) was used to fit the statistical predictive model, and the other (model testing data) was used to test the model and determine the sensitivity and specificity of the predictor for this test run. The test run was repeated 20 times for improved stability [7]. The whole process was performed automatically used our developed codes and the R Package. The optimal sensitivity and specificity (the largest summation of the two values) of these parameters and their area under the receiver operating characteristic curve (AUC) were determined.

## 3. Results

The differences of 1G and 2G models were presented first, then the differences between the two patient groups using 2G models. Post PVR outcome prediction results were presented in 3.10.

### 3.1. Terminologies

The purpose of this paper is to introduce the new model with 2 zero-load geometries (2G model), compare the results with our previous model which used 1 zero-load geometry (1G model), and then use the 2G models of the selected 16 ToF patients to investigate possible associations between morphological and mechanical parameters and post PVR surgical outcome. For the 1G model, results at begin-filling (BF) and begin-ejection (BE) corresponding to minimum and maximum pressure and RV volume were obtained for comparison. For the 2G model, results at begin-filling (BF), end-filling (EF), begin-ejection (BE), and end-ejection (EE) were obtained for comparison. The traditional end-systole, end-diastole, and begin-diastole conditions correspond to our begin-ejection, end-ejection, end-filling, and begin-filling, respectively. They may be used interchangeably as needed.

### 3.2. Begin-ejection stress from the 2G model was 28% higher than that from the 1G model

**Fig 5:**
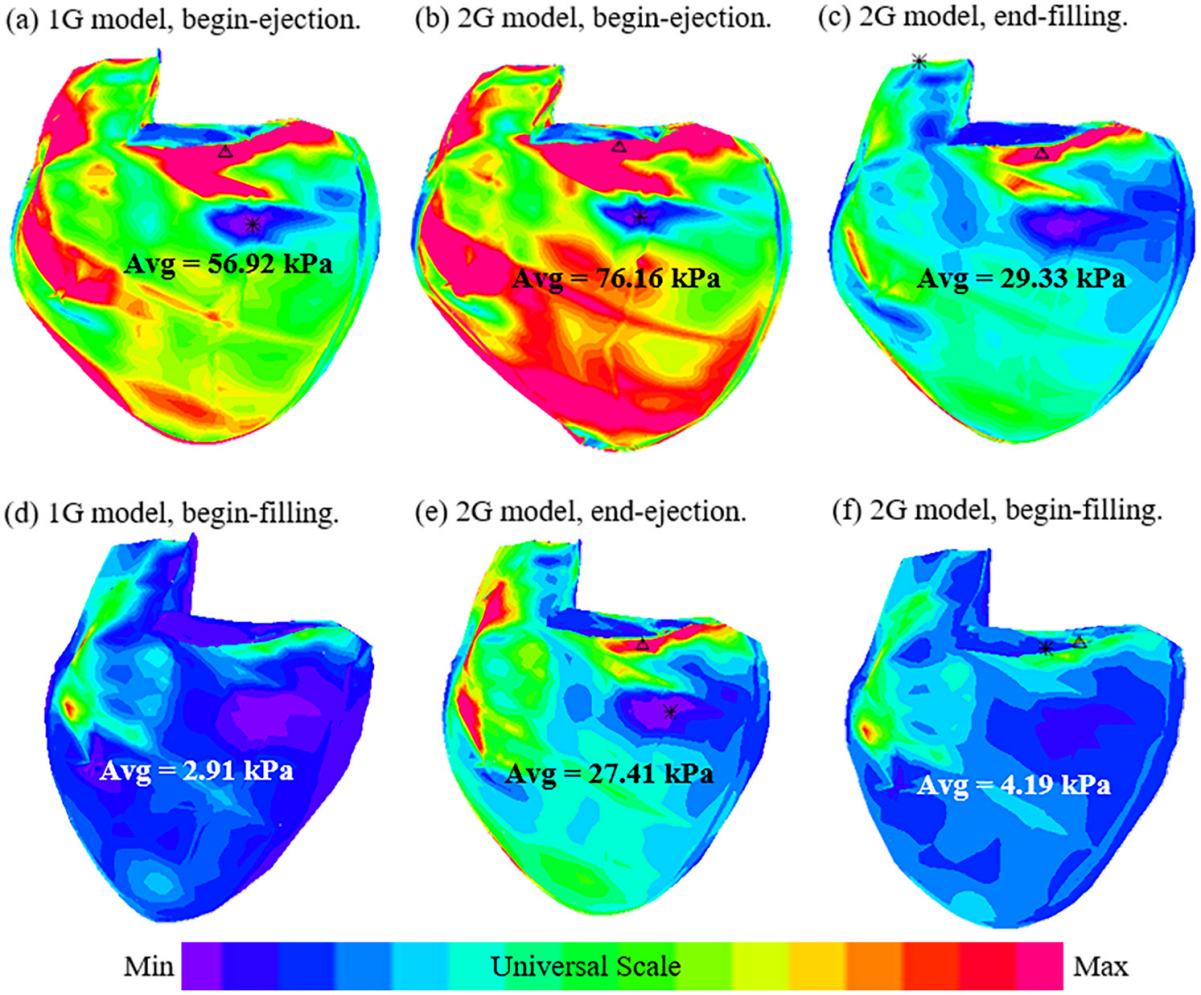
Stress plots from 1G and 2G models showing large differences. (a) 1G model, begin-ejection; (b) 2G model, begin-ejection; (c) 2G model, end-filling; (d) 1G model, begin-filling; (e) 2G model, end-ejection; (f) 2G model, begin-filling. Note: (a)-(c) all with maximum RV volume; (d)-(f) all with minimum RV volume.

Table 2 summarizes the average stress values of the 16 patients from the 2 models. Fig 5 shows the stress plots from the 2 models using the patient given by Fig 1 as an example. According to the total average values in Table 2, begin-ejection stress from the 2G model was 28% higher than that from the 1G model (108.4 kPa vs. 84.7 kPa). The begin-filling stress values from the two models were about the same (7.17 kPa vs. 7.32 kPa, 2% difference).

**Table 2:** Comparison of average stress results from the new (2G) and old (1G) models. BF: Begin-Filling; BE: Begin-Ejection; EF: End-Filling; EE: End-Ejection.

	1G Model		2G Model			
Patient	1G-BF	1G-BE	2G-BF	2G-EF	2G-BE	2G-EE
1	2.914	56.92	4.19	29.32	76.16	27.41
2	9.034	65.35	8.66	43.46	109.90	29.65
3	1.551	41.03	2.62	21.21	58.93	17.46
4	3.655	64.06	5.53	33.56	89.91	30.62
5	9.564	82.41	9.58	55.91	127.76	30.45
6	2.314	61.89	2.59	34.02	92.17	16.02
7	2.160	33.45	2.09	17.13	46.03	9.82
8	2.042	41.97	2.25	21.68	62.14	13.19
9	7.880	172.05	7.43	57.61	161.20	35.88
10	13.229	82.90	13.19	52.42	121.48	36.46
11	15.832	82.42	16.63	50.78	109.86	46.13
12	2.638	83.11	2.86	56.68	146.04	25.21
13	17.799	191.73	10.30	59.89	151.85	33.71
14	3.232	65.36	4.75	34.79	89.98	24.25
15	15.027	154.31	13.90	77.69	182.61	53.04
16	8.307	76.18	8.17	43.40	108.53	32.93
**Ave**	**7.323**	**84.70**	**7.17**	**43.10**	**108.41**	**28.89**

### 3.3. 2G model provides end-systole and end-diastole stress conditions

It should be noted that the 2G model now provides end-systole and end-diastole stress conditions which were not available from 1G model. The right ventricles had the same volume at end-diastole and begin-systole, but RV begin-systole (peak-systole) stress average value was 151.5% higher than the end-diastole average stress (108.41 kPa vs. 43.10 kPa). Similarly, the right ventricles had their minimum volumes at both end-systole and begin-diastole, but RV end-systole stress average value was 300% higher than the begin-diastole average stress (28.89 kPa vs. 7.17 kPa). These stress conditions are of fundamental importance for many cardiovascular investigations.

### 3.4. Begin-systole strain from the 2G model was 39.6% higher than that from the 1G model

**Fig 6:**
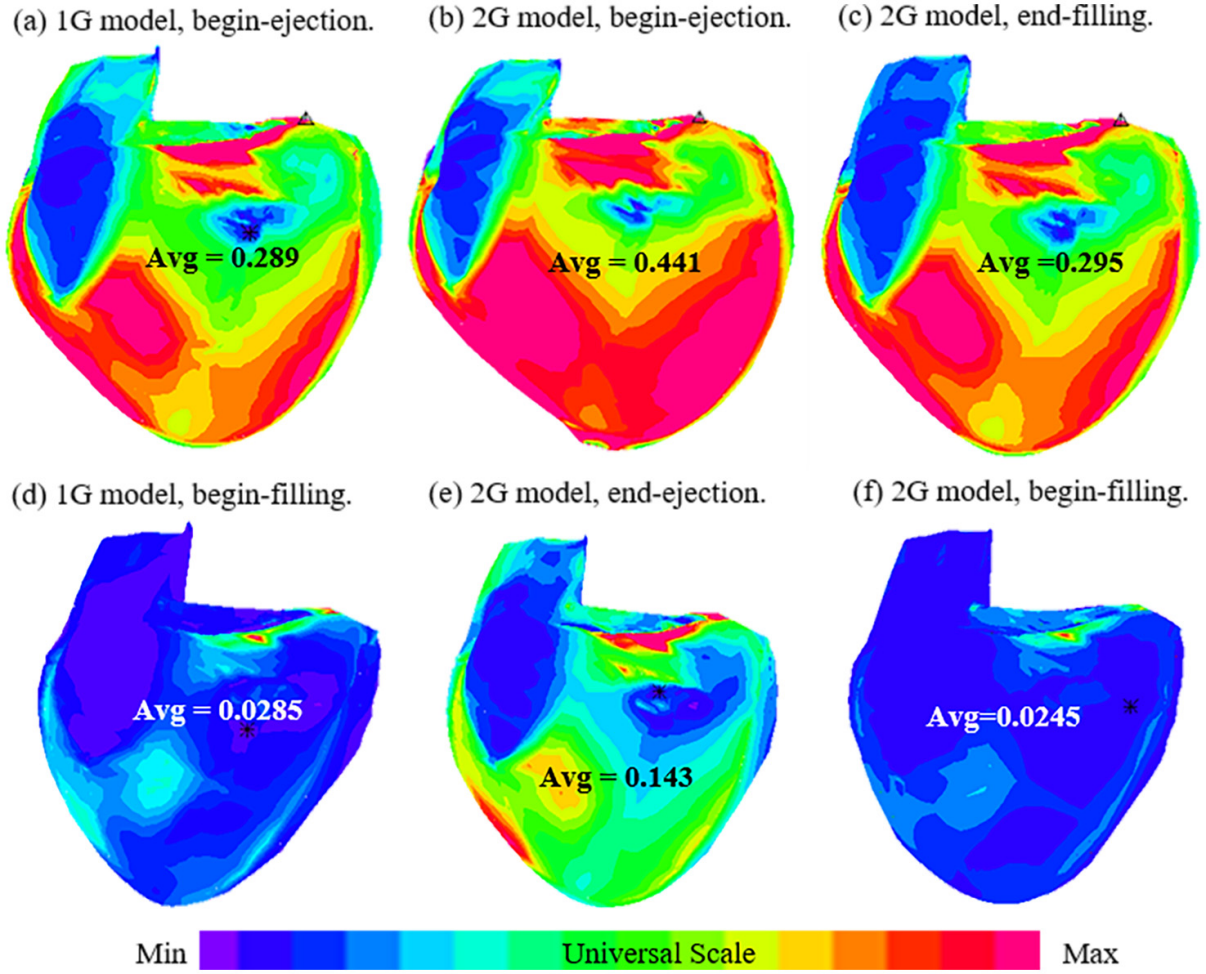
Strain plots from 1G and 2G models showing large differences. (a) 1G model, begin-ejection; (b) 2G model, begin-ejection; (c) 2G model, end-filling; (d) 1G model, begin-filling; (e) 2G model, end-ejection; (f) 2G model, begin-filling. Note: (a)-(c) all with maximum RV volume; (d)-(f) all with minimum RV volume.

**Fig 7:**
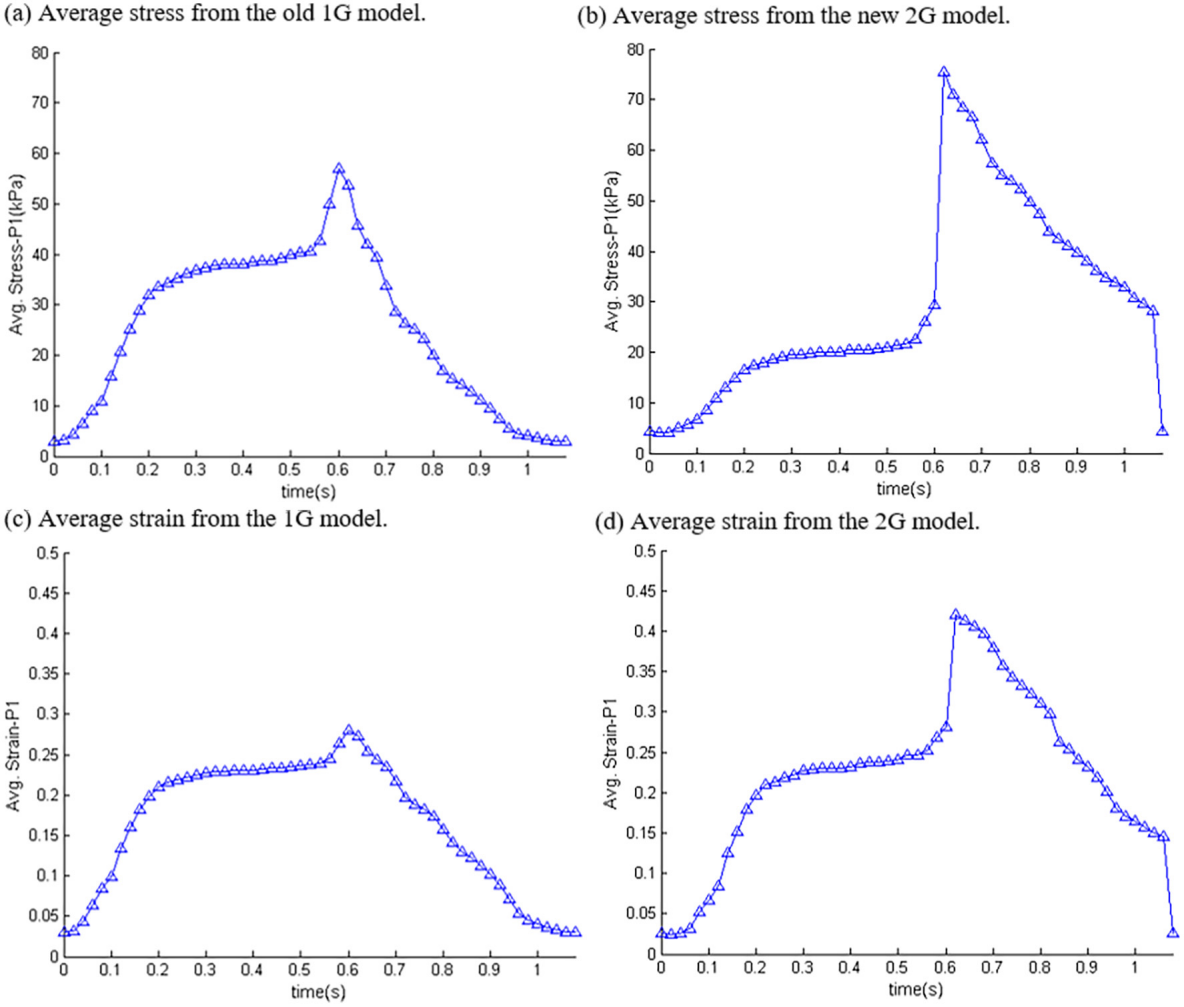
Stress and strain variations (average value on the inner RV surface) in one cardiac cycle from a TOF patient showing the difference between the two models. Sudden increase at the end of diastole and sudden decrease at the end of systole reflected our omission of the two isovolumic phases. (a) Average stress from the old 1G model; (b) average stress from the new 2G model; (c) Average strain from the 1G model; (d) average strain from the 2G model.

Table 3 summarizes the average strain values of the 16 patients from the 2 models. Fig 6 shows the strain plots from the 2 models using the same patient as Fig 5. Fig 7 gives plots of average stress/strain variations in a cardiac cycle from both models. According to the total average values in Table 3, begin-systole strain from the 2G model was 39.6% higher than that from the 1G model (0.606 vs. 0.434). The begin-diastole strain value from the 2G model was 23% lower than that from 1G model (0.048 vs. 0.062).

**Table 3:** Comparison of average strain results from the 1G and 2G models.

	1G Model		2G Model			
Patient	1G-BF	1G-BE	2G-BF	2G-EF	2G-BE	2G-EE
1	0.028	0.289	0.024	0.295	0.441	0.143
2	0.144	0.425	0.115	0.495	0.698	0.262
3	0.029	0.327	0.042	0.330	0.498	0.159
4	0.053	0.356	0.087	0.357	0.534	0.219
5	0.033	0.444	0.036	0.496	0.653	0.124
6	0.017	0.484	0.012	0.480	0.663	0.109
7	0.016	0.463	0.011	0.449	0.658	0.100
8	0.031	0.401	0.016	0.367	0.565	0.119
9	0.091	0.662	0.056	0.588	0.783	0.212
10	0.137	0.489	0.136	0.494	0.649	0.273
11	0.038	0.230	0.040	0.231	0.373	0.153
12	0.010	0.416	0.009	0.510	0.672	0.091
13	0.160	0.658	0.030	0.500	0.743	0.159
14	0.012	0.442	0.017	0.440	0.621	0.101
15	0.142	0.515	0.110	0.463	0.657	0.250
16	0.044	0.335	0.024	0.304	0.481	0.142
**Ave**	**0.062**	**0.434**	**0.048**	**0.425**	**0.606**	**0.164**

### 3.5. 2G model provides end-systole and end-diastole strain conditions not available from 1G model

Table 3 shows that RV peak-systole strain average value was 42.6% higher than the end-diastole average strain (0.606 vs. 0.425), while the ventricles had their maximum volumes under both conditions. Similarly, the right ventricles had their minimum volumes at both end-systole and begin-diastole, but RV end-systole strain average value was 242% higher than the begin-diastole average strain (0.164 vs. 0.048). We are able to get those values because we used different zero-load geometries for diastole and systole phases, respectively.

### 3.6. Comparison of RV wall thickness and curvatures between the new and old models

Fig 5 demonstrated that both new and old models matched CMR measured RV volumes very well. Table 4 gave RV wall thickness and curvature results from the two models. The begin-diastole RV wall thickness from the new and old models were 0.575 cm and 0.564 cm, respectively (2% difference). The begin-systole RV wall thickness from the new and old models were 0.491 cm and 0.505 cm, a 3% difference. The begin-diastole RV circumferential curvatures from the new and old models were 0.638 1/cm and 0.630 1/cm, respectively (1% difference). The begin-systole RV circumferential curvatures from the new and old models were 0.524 1/cm and 0.514 1/cm, a 2% difference. For the longitudinal curvature, the begin-diastole and begin-systole values (1.216, 1.186) from the old model were very close to the begin-diastole and end-diastole values (1.193, 1.155) from the new model. The peak-systole and end-systole longitudinal curvatures from the new models were 1.263 1/cm and 1.389 1/cm, respectively. The higher longitudinal curvatures in the systole phase were due to the larger shrinkage in the longitudinal direction linked to our selected fiber orientations.

**Table 4:** Comparison of RV wall thickness and curvatures results from the 1G and 2G models.

	1G Model		2G Model			
Patient	1G-BF	1G-BE	2G-BF	2G-EF	2G-BE	2G-EE
RV Wall Thickness (cm)						
16P	0.564	0.505	0.575	0.519	0.491	0.525
Circumferential Curvature (1/cm)						
16P	0.630	0.514	0.638	0.516	0.524	0.618
Longitudinal Curvature (1/cm)						
16P	1.216	1.186	1.193	1.155	1.263	1.389

### 3.7. Statistical significance of the reported model differences

Pairwise T-test and Linear Mixed Effect (LME) model were used to determine if the differences from the new and old models were statistically significant with the dependence of the pair-wise observations the clustering effects taking into consideration. Patients were assumed independent. The p-values of our comparisons are given in Table 5, which indicates that the differences > 5% reported were statistically significant.

**Table 5:** P-values of model result comparisons using pairwise T-test and Linear Mixed Effect (LME) models. p = 0.00000 indicates that p < 0.00001. Begin-ejection = begin-systole; end-ejection = end-systole in 2G model.

*Parameter*	*1G Model*	*2G Model*	*p-value (Pairwise t-Test)*	*p-value (LME Model)*
WT	Begin-Filling	Begin-Filling	0.00000	0.00000
C-cur	Begin-Filling	Begin-Filling	0.00014	0.00014
L-cur	Begin-Filling	Begin-Filling	0.20601	0.20600
Stress	Begin-Filling	Begin-Filling	0.84585	0.84585
Strain	Begin-Filling	Begin-Filling	0.00001	0.00001
WT	Begin-Filling	End-Ejection	0.00000	0.00000
C-cur	Begin-Filling	End-Ejection	0.00591	0.00591
L-cur	Begin-Filling	End-Ejection	0.00297	0.00297
Stress	Begin-Filling	End-Ejection	0.00000	0.00000
Strain	Begin-Filling	End-Ejection	0.00000	0.00000
WT	Begin-Ejection	End-Filling	0.00000	0.00000
C-cur	Begin-Ejection	End-Filling	0.12193	0.12193
L-cur	Begin-Ejection	End-Filling	0.19087	0.19087
Stress	Begin-Ejection	End-Filling	0.00000	0.00000
Strain	Begin-Ejection	End-Filling	0.22070	0.22070
WT	Begin-Ejection	Begin-Ejection	0.00000	0.00000
C-cur	Begin-Ejection	Begin-Ejection	0.01045	0.01045
L-cur	Begin-Ejection	Begin-Ejection	0.01613	0.01613
Stress	Begin-Ejection	Begin-Ejection	0.00000	0.00000
Strain	Begin-Ejection	Begin-Ejection	0.00000	0.00000

### 3.8. Correlations between RV EF change (ΔEF) and the morphological and mechanical stress/strain parameters

Correlation analyses were performed to determine whether changes in RV EF from pre- to post-PVR were associated with RV volume, WT, C-curvature, L-Curvature, or stress/strain data and results are given in Table 6. Using the 2G model results, RV EF change correlated negatively with stress (r = -0.609, P = 0.012) and with pre-PVR RV end-diastole volume (r = -0.60, P = 0.015), but did not correlate with WT, C-curvature, L-curvature, or strain.

**Table 6:** Summary of geometric and stress/strain parameters averaged in each patient at begin-ejection and their correlations with RVEF change.

	ΔEF (%)	WT (cm)	C-Cur (1/cm)	L-Cur (1/cm)	RV EDV (mL)	Stress (kPa)	Strain
Group 1	1.4	0.37	0.50	1.52	406.9	76.2	0.44
	-3.4	0.35	0.40	0.77	328.8	109.9	0.70
	-2.6	0.59	0.38	1.15	408.8	58.9	0.50
	-2.9	0.45	0.55	1.60	364.6	89.9	0.53
	4.0	0.47	0.44	0.90	323.3	127.8	0.65
	5.6	0.48	0.50	1.23	204.0	92.2	0.66
	6.6	0.40	0.55	2.19	193.7	46.0	0.66
	2.0	0.51	0.53	1.83	188.3	62.1	0.56
Mean ± SD	1.34 ±3.9	0.45 ±0.08	0.48 ±0.07	1.40 ±0.48	302.3 ±93.7	82.9 ±27.5	0.59 ±0.09
Group 2	-18.0	0.45	0.43	0.81	385.1	161.2	0.78
	-8.4	0.39	1.38	1.40	204.2	121.5	0.65
	-15.2	0.72	0.37	0.65	665.1	109.9	0.37
	-7.0	0.75	0.48	0.75	334.8	146.0	0.67
	-5.0	0.49	0.46	0.99	277.2	151.8	0.74
	-9.5	0.40	0.64	1.71	365.0	90.0	0.62
	-12.3	0.47	0.45	1.36	299.0	182.6	0.66
	-13.4	0.56	0.32	1.34	571.1	108.5	0.48
Mean ± SD	-11.1 ±4.4	0.53 ±0.14	0.57 ±0.34	1.13 ±0.38	387.4 ±154.8	133.9 ±31.4	0.62 ±0.14
*R		-0.313	0.012	0.433	-0.60	-0.608	0.033
*P		0.239	0.965	0.094	0.015	0.012	0.903

* R- and P-values are for the correlations between change in RV EF and geometric and stress/strain data. All values are begin-ejection values. Abbreviations: WT, wall thickness; C-Cur, circumferential curvature; L-Cur, longitudinal curvature; RV, right ventricle.

### 3.9. Group comparison: RV stress of Group 2 was much higher than that of Group 1

Table 7 summarizes the comparison of RV WT, C-curvature, L-curvature, and stress and strain values between the outcome groups at begin-filling, end-filling, begin-ejection, and end-ejection showing stress is the only parameter with significant difference between the two groups. At begin-ejection, mean RV stress of Group 2 was 57.4% higher than that of Group 1 (130.1 ±60.7 vs. 82.7±38.8 kPa; P = 0.0042). Differences at other three time points were similar. It should be noted that stress was the only parameter that showed significant differences between the two groups.

**Table 7:** Comparison of RV wall thickness, circumferential and longitudinal curvature and stress/strain between Group 1 and Group 2 at begin-filling, end-filling, begin-ejection, and end-ejection showing stress is the only parameter with significant difference between the two groups.

	Begin-Filling (Min Volume and Pressure)				End-Filling			
	Group 1	Group 2	%	P-value	Group 1	Group 2	%	P-value
WT (cm)	0.52±0.09	0.63±0.16	18.2	0.1730	0.48±0.08	0.56±0.15	18.6	0.1870
C-Cur (1/cm)	0.60±0.10	0.69±0.35	13.7	0.5421	0.48±0.07	0.56±0.33	17.2	0.5111
L-Cur (1/cm)	1.33±0.56	1.05±0.34	-20.8	0.2525	1.25±0.47	1.06±0.38	-14.9	0.401
Stress (kPa)	**4.69±2.98**	**9.65±4.73**	**105.9**	**0.0277**	**32.0±12.9**	**54.2±12.6**	**69.0**	**0.0037**
Strain	0.04±0.04	0.05±0.05	23.0	0.6497	0.41±0.08	0.44±0.12	8.0	0.5287
	Begin-Ejection (max V and pressure)				End-Ejection			
	Group 1	Group 2		P-value	Group 1	Group 2		P-value
WT (cm)	0.45±0.08	0.53±0.14	16.8	0.2059	0.48±0.08	0.57±0.15	18.1	0.1767
C-Cur (1/cm)	0.48±0.07	0.57±0.34	17.6	0.5101	0.57±0.09	0.67±0.36	16.9	0.4832
L-Cur (1/cm)	1.40±0.48	1.13±0.38	-19.5	0.2284	1.59±0.74	1.18±0.37	-25.8	0.1899
Stress (kPa)	**82.9±27.5**	**133.9±31.4**	**61.6**	**0.0039**	**22.8±8.6**	**36.0±9.7**	**64.7**	**0.0083**
Strain	0.59±0.09	0.62±0.13	5.7	0.5698	0.15±0.06	0.17±0.07	11.8	0.5662

Notes. Data is based on slice mean values (see Methods for details). Values are expressed as mean ± standard deviation. Abbreviations as before.

### 3.10. Combination of morphological and mechanical stress parameters may provide better prediction for post PVR outcome

The logistic regression method was applied to all 255 possible combinations of the 8 candidate predictors to calculate their prediction accuracy for patient’s group category. The 8 predictors are WT, C-cur, L-cur, RV volume, and stress at begin-ejection, plus three stress variations from one time point to another: StressE-D is stress difference between begin-ejection and end-filling; StressE-F is stress difference between begin-ejection and end-ejection; and StressE-C is stress difference between begin-ejection and begin-filling. Table 8 shows the 6 best combinations (out of 255) of RV parameters that correctly assigned patients to their ultimate outcome group and the prediction accuracy and ranking of the single predictions. Pre-PVR RV stress at begin-ejection was the best single predictor among the 8 individual parameters with an area under the ROC curve of 0.782. The best combination of parameters was C-cur + RV volume + StressE-F with an area under the ROC curve of 0.855.

**Table 8:** Prediction sensitivity, specificity, and AUC values RV parameters for outcome group prediction by the logistic regression method.

Parameter	Prob. CutOffs	Sensiti-vity	Specifi-city	Sensi+ Speci-	AUC	AUC Average	95% Confidence Interval		Rank
C-Cur+V+ StressE-F	0.996	0.681	0.938	1.619	0.854	0.855	0.843	0.861	1
C-Cur+V+ StressE-D	0.999	0.688	0.931	1.619	0.830	0.853	0.847	0.861	2
C-Cur+L-Cur+ V+ StressE-F	0.032	0.738	0.869	1.606	0.857	0.841	0.835	0.859	3
C-Cur+V+ StressE-C	0.949	0.713	0.919	1.631	0.864	0.841	0.836	0.845	4
C-Cur+V+ StressE	0.998	0.650	0.950	1.600	0.854	0.837	0.832	0.844	5
C-Cur+StressE-F +V+StressE-D	0.023	0.825	0.794	1.619	0.865	0.816	0.811	0.841	6
Stress E	0.446	0.800	0.781	1.581	0.782	0.769	0.762	0.771	25
Stress E-D	0.445	0.738	0.738	1.475	0.765	0.756	0.749	0.759	32
Stress E-F	0.504	0.681	0.719	1.400	0.721	0.747	0.742	0.757	37
Stress E-C	0.378	0.813	0.706	1.519	0.749	0.747	0.732	0.753	38
WT	0.552	0.363	0.800	1.163	0.571	0.526	0.522	0.532	243
L-Cur	0.352	0.825	0.281	1.106	0.534	0.525	0.520	0.528	244
V	0.379	0.781	0.300	1.081	0.502	0.504	0.497	0.510	247
C-Cur	0.859	0.044	0.975	1.019	0.410	0.399	0.394	0.413	255

AUC average and 95% confidence interval are based on 100 rounds of 20 repeats of the cross-validation. Abbreviations as before. Stress E = stress at begin-ejection.

## 4. Discussions

It should be noted that our 1G model starts from RV minimum volume, with minimum pressure, stress and strain conditions.

For ventricle modeling, Peskin pioneered heart modeling effort and simulated blood flow in a pumping heart with his immersed boundary method [21]. McCulloch et al. and Hunter et al. conducted comprehensive investigations for cardiac mechanics, which included passive and active ventricle modeling, the Physiome Project and the Continuity package [2–3]. Kerckhoffs et al. introduced a multi-scale approach starting from the cellular scale and building to the tissue, organ and system scales [22–23]. As heart contraction is triggered by electrical activation, Pfeiffer et al. investigated cardiac mechanics with electromechanical coupling and mechanoelectric feedback [24]. Guccione et al. presented a detailed left ventricle active contraction model with parameter values determined from canines [25]. Costa et al. studied ventricle laminar fiber architecture and 3D systolic mechanics using canine model [26]. Humphrey et al. and Novak et al. investigated ventricle tissue material properties using animal tissues [15–16]. Gan et al. used MRI-based left ventricle models and Cine-MRI to perform strain rate analysis and indicated that strain rate may be used to differentiate diabetic patients from normal controls [27]. Early magnetic resonance imaging (MRI)-based ventricle models were introduced by Saber et al. for mechanical analysis and investigations [28]. Choi et al. used MRI-based models which coupled electromechanics with hemodynamics to compare normal and diseased canine left ventricle cardiac functions [29]. Das et al., used patient-specific phase-contrast MRI (PC-MRI) to quantify flow velocity boundary conditions for ventricle models to get better flow predictions [30]. Asner et al. used tagged MRI to estimate passive and active properties in the human heart [31]. Nguyen et al. patient-specific CFD models to investigate left ventricle (LV) diastolic dysfunctions [32]. Krishnamurthy et al. described a unique approach mapping a 3D bi-ventricular model obtained from a fixed heart to patient-specific geometric models using large deformation diffeomorphic mapping [33]. These methods were tested in five heart failure patients and their results showed good agreement with measured echocardiographic and global functional parameters (ejection fraction and peak cavity pressures). Fomovsky et al. used models in design of mechanical therapies for myocardial infarction [34]. Holmes et al. indicated that image-based cardiac mechanical models could provide useful information for clinical and surgical applications [35]. In our previous papers, patient-specific MRI-based computational right ventricle/left ventricle (RV/LV) models with fluid-structure interactions were introduced to assess outcomes of various RV reconstruction techniques with different scar tissue trimming and patch sizes [4–7].

### 4.1. Significance of the new 2G models

Correct stress/strain calculation is of fundamental importance for many cardiovascular research where mechanical forces play a role. Ventricle remodeling, disease development, tissue regeneration, patient recovery after surgery and many other cell activities are closely associated with ventricle mechanical conditions. The 2G modeling approach is setting up the right stage for diastole and systole stress/strain calculations using proper zero-load geometries. 1G models do not use different reference geometries for systole and diastole phases, therefore have difficulties in giving right strain calculations. It should be noted that direct measurements of stress, strain, and zero-load sarcomere length are either extremely difficult or even impossible. Even by using tagging, the strain determined uses in vivo references and could not account for zero-stress SL changes. Actual ventricle contraction and relaxation are very complex. Our model is only a first-order approximation, an improvement over the 1G models. Lack of in vivo data and model construction cost are also considerations. Data from the literature or from ex vivo experiments have to be used to complete the computational models. We are in need of patient-specific data such as fiber orientation, sarcomere length contraction rate, regional material properties, etc.

### 4.2. Predictors for post PVR outcome

Survival of patients with tetralogy of Fallot (TOF) has steadily increased since the introduction of open-heart surgery, with operative mortality currently <2% [36]. Survival past the first two decades of life has also improved with recent reports showing a 30-year survival rate nearing 90% [37]. Since this operation was first performed in the mid-1950s, a conservative estimate projects that the number of survivors of TOF repair in the United States exceeds 100,000 and increases by 3,000–4,000 patients every year [38]. As a result of the surgical reconstruction of the right ventricular (RV) outflow tract and other operative sequelae, patients are exposed to chronic pulmonary regurgitation that leads to progressive RV dilatation and dysfunction. The current surgical approach to address chronic pulmonary regurgitation includes pulmonary valve replacement/insertion (PVR) with or without RV remodeling. However, while most patients demonstrate a variable degree of decrease in RV size, many do not experience an improvement in RV function and some show a decline after PVR [38–39]. In our previously reported randomized clinical trial of surgical remodeling of the RVOT in 64 patients with repaired TOF undergoing PVR, using available clinical data and CMR, we found no significant difference between groups with and without RV remodeling in post PVR RV EF changes [8]. It has remained unclear why some patients had experienced an improvement in RV EF whereas in others RV function had deteriorated. Our modeling added mechanical stress analysis to this study and the identified predictors provided a potential approach to identify patients and factors for possible surgical outcome improvements.

### 4.3. Model limitations and future directions

Several improvements can be added to our models in the future for better accuracy and applicability: a) fluid-structure interaction to obtain both flow and structural stress/strain information for complete mechanical analysis; b) direct measurements of tissue mechanical properties which will be very desirable for improved accuracy of our models; c) patient-specific fiber orientations and better estimate of zero-stress sarcomere fiber contraction rate; d) valve mechanics would enable us to investigate regurgitation and other valve-related diseases.

Small sample size (n = 16) is a limitation for the prediction method. Repeated cross-validation was used which is a standard technique to reduce errors and improve prediction stability, especially when sample size is relatively small [19, 20]. Our prediction method was also used in our previous one-geometry paper [7] where consistent results were reported. It should be noted that the only real solution for the small sample size issue is to increase the patient numbers. Since getting new patients to obtain data with follow-up data after surgery is extremely difficult and requires time and resources, and model construction takes long time (each model takes 2–4 weeks to construct and adjust; each patient needs practically 3 models: 1G, 2G systole and 2G diastole), adding more patients will be our future effort.

## References

[1] Goetz WA, Lansac E, Lim HS, Weber PA, Duran CM. Left ventricular endocardial longitudinal and transverse changes during isovolumic contraction and relaxation: a challenge. Am J Physiol Heart Circ Physiol. 2005 7;289(1):H196–201. 15708963 10.1152/ajpheart.00867.2004

[2] McCulloch AD, Waldman L, Rogers J, Guccione JM. Large-scale finite element analysis of the beating heart, Critical Rev. in Biomed Eng, 1992; 20(5,6): 427–449. 1486784

[3] Hunter PJ, Pullan AJ, Smaill BH. Modeling total heart function, Annu Rev Biomed Eng., 2003; 5:147–77. 14527312 10.1146/annurev.bioeng.5.040202.121537

[4] Tang D, Yang C, Geva T, del Nido PJ. Image-Based Patient-Specific Ventricle Models with Fluid-Structure Interaction for Cardiac Function Assessment and Surgical Design Optimization, Progress in Pediatric Cardiology, 30:51–62, 2010 21344066 10.1016/j.ppedcard.2010.09.007 PMC3041970

[5] Tang D, Yang C, Geva T, Gaudette G, del Nido PJ., Multi-Physics MRI-Based Two-Layer Fluid-Structure Interaction Anisotropic Models of Human Right and Left Ventricles with Different Patch Materials: Cardiac Function Assessment and Mechanical Stress Analysis, Computers & Structures, 89, 1059–1068, 2011. 21765559 10.1016/j.compstruc.2010.12.012 PMC3134331

[6] Tang D, Yang C, Geva T, del Nido PJ. Patient-specific MRI-based 3D FSI RV/LV/Patch models for pulmonary valve replacement surgery and patch optimization, J. of Biomech. Engineering, 2008;130(4) 041010. 10.1115/1.2913339 PMC2918812 18601452

[7] Tang D, Yang C, Del Nido PJ, Zuo H, Rathod RH, Huang X, Gooty V, Tang A, Billiar KL, Wu, Geva T. Mechanical stress is associated with right ventricular response to pulmonary valve replacement in patients with repaired tetralogy of Fallot. J Thorac Cardiovasc Surg. 2015 10 3 pii: S0022-5223(15)01815-2. 10.1016/j.jtcvs.2015.09.106 PMC4761474 26548998

[8] Geva T, Gauvreau K, Powell AJ, Cecchin F, Rhodes J, Geva J, del Nido P. Randomized trial of pulmonary valve replacement with and without right ventricular remodeling surgery, Circulation. 2010 9 14;122(11 Suppl):S201–8. 10.1161/CIRCULATIONAHA.110.951178 20837914 PMC2943672

[9] Geva T, Is MRI the preferred method for evaluating right ventricular size and function in patients with congenital heart disease?: MRI is the preferred method for evaluating right ventricular size and function in patients with congenital heart disease, Circ Cardiovasc Imaging. 2014;7:190–197. 10.1161/CIRCIMAGING.113.000553 24449548 PMC4006374

[10] Geva T, Repaired tetralogy of Fallot: the roles of cardiovascular magnetic resonance in evaluating pathophysiology and for pulmonary valve replacement decision support, Journal of Cardiovascular Magnetic Resonance 2011, 13:9. 10.1186/1532-429X-13-9 PMC3036629 21251297

[11] Sanchez-Quintana D, Anderson R, Ho SY, Ventricular myoarchitecture in tetralogy of Fallot, Heart, 1996;76:280–286. 8868990 10.1136/hrt.76.3.280 PMC484521

[12] Sacks MS, Chuong CJ, Biaxial mechanical properties of passive right ventricular free wall myocardium, J Biomech Eng, 1993; 115:202–205. 8326727 10.1115/1.2894122

[13] Valdez-Jasso D, Simon MA, Champion HC, Sacks MS. A murine experimental model for the mechanical behaviour of viable right-ventricular myocardium. J Physiol. 2012 9 15;590(Pt 18):4571–84. 10.1113/jphysiol.2012.233015. Epub 2012 Jul 30. 22848044 PMC3477758

[14] Billiar KL, Sacks MS. Biaxial mechanical properties of the natural and glutaraldehyde treated aortic valve cusp—Part I: Experimental results. J Biomech Eng. 2000;122(1):23–30. 10790826 10.1115/1.429624

[15] Humphrey JD, Strumpf RK, Yin FC. Biaxial mechanical behavior of excised ventricular epicardium. Am J Physiol. 1990 7;259(1 Pt 2):H101–8. 2375396 10.1152/ajpheart.1990.259.1.H101

[16] Novak VP, Yin FC, Humphrey JD. Regional mechanical properties of passive myocardium. J Biomech. 1994 4;27(4):403–12. 8188721 10.1016/0021-9290(94)90016-7

[17] Tang D, Yang C, Geva T, del Nido PJ. Right ventricular local longitudinal curvature as a marker and predictor for pulmonary valve replacement surgery outcome: an initial study based on preoperative and postoperative cardiac magnetic resonance data from patients with repaired tetralogy of Fallot. J Thorac Cardiovasc Surg. 2014 1;147(1):537–8. 10.1016/j.jtcvs.2013.08.054 24100105 PMC3957093

[18] Hastie T, Tibshirani R, Friedman J, The Elements of Statistical Learning: Data Mining, Inference, and Prediction, Second Edition, New York, Springer, 2009.

[19] Kim JH, Estimating classification error rate: Repeated cross-validation, repeated hold-out and bootstrap. Computational Statistics & Data Analysis, 2009; 53(11):3735–3745.

[20] Braga-Neto UM, Dougherty ER, Is cross-validation valid for small-sample microarray classification? Bioinformatics, 2004; 20(3):374–380. 14960464 10.1093/bioinformatics/btg419

[21] Peskin CS. Numerical analysis of blood flow in the heart. J Com Phys. 25:220–252, 1977.

[22] Kerckhoffs RC, Neal ML, Gu Q, Bassingthwaighte JB, Omens JH, McCulloch AD. Coupling of a 3D finite element model of cardiac ventricular mechanics to lumped systems models of the systemic and pulmonic circulation. Ann Biomed Eng. 2007 1;35(1):1–18. 17111210 10.1007/s10439-006-9212-7 PMC2872168

[23] Kerckhoffs RC, Campbell SG, Flaim SN, Howard EJ, Sierra-Aguado J, Mulligan LJ, McCulloch AD. Multi-scale modeling of excitation-contraction coupling in the normal and failing heart. Conf Proc IEEE Eng Med Biol Soc. 2009;2009:4281–2. 10.1109/IEMBS.2009.5332708 19963818 PMC2896048

[24] Pfeiffer E, Tangney J, Omens J, McCulloch AD, Biomechanics of Cardiac Electromechanical Coupling and Mechanoelectric Feedback. J Biomech Eng. 2014, 136(2):021007 10.1115/1.4026221 24337452 PMC4023651

[25] Guccione JM, Waldman LK, McCulloch AD. Mechanics of active contraction in cardiac muscle: Part II—Cylindrical models of the systolic left ventricle, J Biomech Eng. 1993;115(1):82–90. 8445902 10.1115/1.2895474

[26] Costa KD, Takayama Y, McCulloch AD, and Covell JW. Laminar fiber architecture and three-dimensional systolic mechanics in canine ventricular myocardium, Am J Physiol. 1999; 276(2 Pt 2):H595–607. 9950861 10.1152/ajpheart.1999.276.2.H595

[27] Gan Y, Chen Q, Zhang S, Ju S, Li ZY. MRI-based strain and strain rate analysis of left ventricle: a modified hierarchical transformation Model, BioMedical Engineering OnLine 2015, 14, S1:S9. 10.1186/1475-925X-14-S1-S9. Epub 2015 Jan 9, 2015. PMC4306125 25602778

[28] Saber NR, Gosman AD, Wood NB, Kilner PJ, Charrier CL, Firman DN. Computational flow modeling of the left ventricle based on in vivo MRI data: initial experience, Annals of Biomed. Engng., 2001; 29:275–283. 10.1114/1.1359452 11339325

[29] Choi YJ, Constantino J, Vedula V, Trayanova N, Mittal R. A new MRI-based model of heart function with coupled hemodynamics and application to normal and diseased canine left ventricles. Front Bioeng Biotechnol. 2015 9 23;3:140 10.3389/fbioe.2015.00140 26442254 PMC4585083

[30] Das A, Wansapura JP, Gottliebson WM, Banerjee RK. Methodology for implementing patient-specific spatial boundary condition during a cardiac cycle from phase-contrast MRI for hemodynamic assessment. Med Image Anal. 2015 1;19(1):121–36. 10.1016/j.media.2014.09.001 25461332

[31] Asner L, Hadjicharalambous M, Chabiniok R, Peresutti D, Sammut E, Wong J, Carr-White G, Chowienczyk P, Lee J, King A, Smith N, Razavi R, Nordsletten D. Estimation of passive and active properties in the human heart using 3D tagged MRI. Biomech Model Mechanobiol. 2015 11 26. [Epub ahead of print] 10.1007/s10237-015-0748-z PMC5021775 26611908

[32] Nguyen VT, Wibowo SN, Leow YA, Nguyen HH, Liang Z, Leo HL. A patient-specific computational fluid dynamic model for hemodynamic analysis of left ventricle diastolic dysfunctions. Cardiovasc Eng Technol. 2015;6(4):412–29. 10.1007/s13239-015-0244-8 26577476

[33] Krishnamurthy A, Villongco CT, Chuang J, Frank LR, Nigam V, Belezzuoli E, Stark P, Krummen DE, Narayan S, Omens JH, McCulloch AD, Kerckhoffs RC. Patient-specific models of cardiac biomechanics. J. Comput. Phys. 2013; 244: 4–21. 23729839 10.1016/j.jcp.2012.09.015 PMC3667962

[34] Fomovsky GM, Macadangdang JR, Ailawadi G, Holmes JW. Model-based design of mechanical therapies for myocardial infarction. J Cardiovasc Transl Res. 2011 2;4(1):82–91. 10.1007/s12265-010-9241-3 Epub 2010 Nov 19. 21088945 PMC3328213

[35] Holmes JW, Costa KD. Imaging cardiac mechanics: what information do we need to extract from cardiac images? Conf Proc IEEE Eng Med Biol Soc. 2006; 1: 1545–1547. 17946901 10.1109/IEMBS.2006.259642

[36] Ooi A, Moorjani N, Baliulis G, Keeton BR, Salmon AP, Monro JL, Haw MP. Medium term outcome for infant repair in tetralogy of Fallot: indicators for timing of surgery. European Journal of Cardio-thoracic Surgery 2006;30:917–922. 17052914 10.1016/j.ejcts.2006.08.022

[37] Chiu SN, Wang JK, Chen HC, Lin MT, Wu ET, Chen CA, Huang SC, Chang CI, Chen YS, Chiu IS, Chen CL, Wu MH. Long-Term Survival and Unnatural Deaths of Patients With Repaired Tetralogy of Fallot in an Asian Cohort. Circulation Cardiovasc Qual Outcomes. 2012;5:120–125. 10.1161/CIRCOUTCOMES.111.963603 22235069

[38] McKenzie ED, Khan MS, Dietzman TW, Guzmán-Pruneda FA, Samayoa AX, Liou A, Heinle JS, Fraser CD Jr. Surgical pulmonary valve replacement: a benchmark for outcomes comparisons. J Thorac Cardiovasc Surg. 2014;148(4):1450–3. 10.1016/j.jtcvs.2014.02.060 24703628

[39] Waien SA, Liu PP, Ross BL, Williams WG, Webb GD, McLaughlin PR. Serial follow-up of adults with repaired tetralogy of Fallot. J Am Coll Cardiol. 1992;20: 295–300. 1634663 10.1016/0735-1097(92)90093-3

